# Evidence against an ecological explanation of the jitter advantage for vection

**DOI:** 10.3389/fpsyg.2014.01297

**Published:** 2014-11-11

**Authors:** Stephen Palmisano, Robert S. Allison, April Ash, Shinji Nakamura, Deborah Apthorp

**Affiliations:** ^1^School of Psychology, University of WollongongWollongong, NSW, Australia; ^2^Department of Electrical Engineering and Computer Science, York UniversityToronto, ON, Canada; ^3^Division of Clinical Psychology, Faculty of Child Development, Nihon Fukushi UniversityNagoya, Japan; ^4^Research School of Psychology, Australian National UniversityCanberra, ACT, Australia

**Keywords:** vection, self-motion, optic flow, ecology, sensory conflict, locomotion, treadmill

## Abstract

Visual-vestibular conflicts have been traditionally used to explain both perceptions of self-motion and experiences of motion sickness. However, sensory conflict theories have been challenged by findings that adding simulated viewpoint jitter to inducing displays enhances (*rather than* reduces or destroys) visual illusions of self-motion experienced by stationary observers. One possible explanation of this jitter advantage for vection is that jittering optic flows are more ecological than smooth displays. Despite the intuitive appeal of this idea, it has proven difficult to test. Here we compared subjective experiences generated by jittering and smooth radial flows when observers were exposed to either visual-only or multisensory self-motion stimulations. The display jitter (if present) was generated in real-time by updating the virtual computer-graphics camera position to match the observer’s tracked head motions when treadmill walking or walking in place, or was a playback of these head motions when standing still. As expected, the (more naturalistic) treadmill walking and the (less naturalistic) walking in place were found to generate very different physical head jitters. However, contrary to the ecological account of the phenomenon, playbacks of treadmill walking and walking in place display jitter both enhanced visually induced illusions of self-motion to a similar degree (compared to smooth displays).

## INTRODUCTION

As we move through the world, we detect our self-motion using multiple senses, including vision, the vestibular system of the inner ear, proprioception, somatosensation, and audition (Gibson, 1966; Dichgans and Brandt, 1978; Howard, 1982; Benson, 1990). The visual and vestibular contributions to this self-motion processing are thought to be especially important (Dichgans and Brandt, 1978; Howard, 1982). The key role that vision plays is clearly demonstrated by the fact that compelling illusions of self-motion can be induced by visual self-motion stimulation alone (known as “vection”; Fischer and Kornmüller, 1930; Tschermak, 1931). Notably, while the visual system is able to detect both constant and accelerating self-motions (based on the optical flow presented to the moving observer), the inertial sensors comprising the vestibular end organs only respond to acceleration (Howard, 1982; Benson, 1990). Given this limitation of the vestibular system, it was long considered that: (a) visually simulated smooth/constant self-motions should induce the strongest vection – since such displays would be expected to produce minimal visual-vestibular conflict in stationary observers; and (b) visually simulated self-acceleration would impair, or possibly even destroy, vection – since the vestibular stimulation normally accompanying this simulated self-motion would be absent (Zacharias and Young, 1981).

Contrary to both of these predictions, we now know that vection can be significantly enhanced by adding a variety of visually simulated self-accelerations to smooth, constant self-motion displays (Palmisano et al., 2000, 2003, 2007, 2008; Palmisano and Chan, 2004; Kim and Palmisano, 2008; Bubka and Bonato, 2010; Nakamura, 2010, 2012; Kim et al., 2012; Apthorp and Palmisano, 2014; Kim and Khuu, 2014). Despite the expected increases in sensory conflict, adding visually simulated viewpoint jitter to optic flow has been shown to significantly decrease vection onset latencies, lengthen vection durations and strengthen vection ratings (e.g., Palmisano et al., 2000; see Palmisano et al., 2011 for a review).

To date, one of the most persistent explanations for this “jitter advantage” for vection is based on the observation that smooth optical/retinal flow rarely occurs in the real world. Until recently, vection was typically induced by displays which simulated constant velocity linear/rotary self-motion along/about a single axis. However, walking and running through the world actually generates complex six degree-of-freedom (6DOF) head movements. In addition to an overall forward displacement, such self-motions also generate random and oscillatory “bob,” “sway,” and “lunge” head displacements, as well as 3D head rotations (Grossman et al., 1988; Cutting et al., 1992; Hirasaki et al., 1999; von Grünau et al., 2007). The end result is a rich multi-axis mix of head motion amplitudes and frequencies. Since head jitter in these situations can be as high as 15 Hz, and can also include linear components, the visual perspective jitter generated by such head movements can only be partially compensated for by eye-movements (Grossman et al., 1989; von Grünau et al., 2007). Accordingly, it has been proposed that self-motion perception might be specialized for (i.e., tuned to) the jittering retinal flow accompanying everyday locomotion^1^. That is, jittering self-motion displays might induce superior vection to smooth, constant velocity self-motion displays because they are more ecological/naturalistic and therefore better matched to visual self-motion processing (e.g., Bubka and Bonato, 2010). In apparent support of this proposal, Bubka and Bonato (2010) reported that movies filmed while walking with a handheld camera induced superior vection to other control movies which were filmed from a rolling cart (the former and latter situations should have produced more naturalistic and minimal/unnatural jitter respectively).

Despite the intuitive appeal of this ‘ecological’ explanation of jitter effects on vection, and Bubka and Bonato’s (2010) observations, it is actually a rather challenging hypothesis to test. Simply comparing the vection enhancements provided by adding ecological and artificially generated display jitter (compared to no jitter or smooth display conditions) does not provide a conclusive test. The artificial jitter stimuli required for this type of study should, by necessity, match the complexity and other characteristics of real head jitter as closely as possible, while still serving as viable control stimuli. However, it is difficult to adjust any one of the multiple display factors thought to make displays more/less ecological, without also changing other, potentially critical, low-level display factors. For example, one cannot create a viable jittering control stimulus by simply scrambling real head position and orientation data, since the resulting frequency spectrum of this artificial jitter would be dramatically different from that of the real head jitter upon which they were based.

In the current study, we attempted to test the ecological account of the jitter advantage by comparing the effects of more and less ecological patterns of simulated viewpoint jitter on vection. In both cases, complex 6DOF visual viewpoint jitter was added to the radially expanding optic flow display which simulated constant velocity forward self-motion. Two types of walking were used to generate this visual jitter: (1) Walking on a motorized treadmill – where participants repeatedly stepped in a forward direction while walking on the moving belt of the treadmill; and (2) Walking in place – where participants repeatedly moved their legs forward and then reversed this leg motion so as to return their feet to their original position on the stationary ground surface. Of these two types of walking, treadmill walking is generally regarded as the more naturalistic (Pozzo et al., 1990; Templeman et al., 1999; Usoh et al., 1999; Yan et al., 2004; Warabi et al., 2005; Ivanenko et al., 2006; Riley et al., 2007; Lee and Hidler, 2008). In fact, treadmill gait appears to be qualitatively and quantitatively similar to overground gait (e.g., Riley et al., 2007), and as a result, head kinematics are similar to those during normal walking (e.g., Pozzo et al., 1990; Lee and Hidler, 2008).

In order to test the ecological account, it was important to first show that the physical characteristics of 6DOF head jitter generated by the “more ecological” treadmill walking and the “less ecological” walking in place conditions differed significantly^2^. Thus we initially conducted a series of head movement analyses that compared the head jitter frequencies and amplitudes generated by the two types of walking. These analyses examined whether the two types of walking produced: (1) different dominant peak head movement amplitudes and frequencies; (2) different distributions of spectral content; and (3) differences in the balance of the motion energy across the three orthogonal axes of head movement.

This study was however primarily interested in whether: (1) the visual jitter generated by the treadmill walking would induce superior vection to that generated by the walking in place; and (2) both types of jittering radial flow would induce superior vection to the smooth radial flow. During the walking trials, head movements generated by either the treadmill walking or walking in place were directly incorporated into the self-motion display in real time (as changes to both the position and orientation of the virtual camera). These jittering radial flow displays were also later played back to participants when they were standing still. Self-motion perceptions in these subject stationary “playback” trials were crucial as they compared the effects of the two visual jitter types on vection (Note: vection is traditionally defined as a visual illusion of self-motion induced in a physically stationary observer). By contrast, the walking conditions allowed us to compare the effects of the two types of visual-and-head jitter on multisensory (i.e., visual + non-visual) perceptions of self-motion for the first time.

## MATERIALS AND METHODS

This study was approved by the University of Wollongong Human Research Ethics Committee (HE10/120) and written consent was obtained from all participants prior to participating in the study.

### PARTICIPANTS

Nineteen Psychology students (15 females and 4 males; mean age = 23.28, SD = 2.87) from the University of Wollongong participated in this experiment. All of these participants had normal or corrected-to-normal vision and no self-reported vestibular or neurological impairments.

### APPARATUS

**FIGURE 1:**
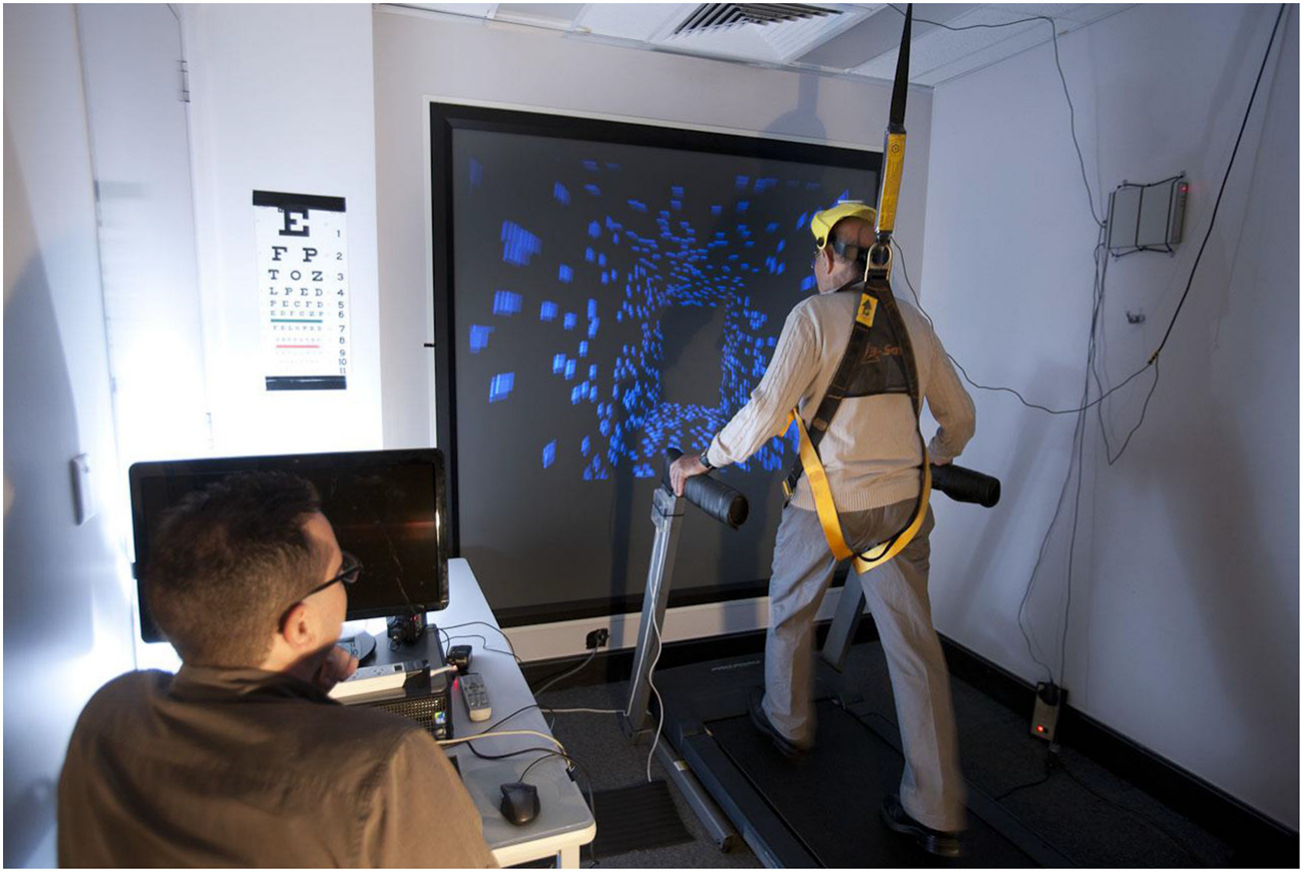
**The general experimental setup.** Please note that moving 3-D clouds of spherical objects were used as the visual stimuli for this experiment (not the tiled virtual corridor which is depicted). All room lighting was turned off during the actual testing.

The general experimental setup is shown in **Figure 1**. The computer-generated self-motion displays were generated on a Dell Optiplex GX620 PC and rear-projected onto a flat projection screen (1.48 m wide × 1.20 m high) by a Mitsubishi Electric (Model XD400U) color DLP data projector. The displayed images subtended a visual area of ∼79° wide by ∼67° high and had a 1024 (horizontal) × 768 (vertical) pixel resolution as well as a refresh rate of 72 Hz. Each display (viewed in an otherwise completely dark room) simulated either a 4 or 5 km/h forward self-motion through a 3D cloud of randomly positioned objects. The cloud – the dimensions of which were simulated to be 1.7 m wide by 2.6 m high by 10.8 m deep – consisted of 1000 blue spheres (300 cd/m^2^) on a black background (0.1 cd/m^2^). On different trials, these self-motion displays were viewed either while standing still, walking in place, or walking forward on the motorized treadmill (ProForm PF 4.0). Participant head position and orientation were continuously recorded during both types of walking trial, via an ultrasonic Logitech 3-D head tracker (see Ash et al., 2013 for details). For safety reasons, participants wore a ceiling mounted B-Safe body harness throughout the entire experiment (during both walking and standing still blocks).

### PROCEDURE

In this experiment, we examined self-motion percepts generated by viewing radially expanding optic flow in the following three situations: (1) treadmill walking, (2) walking in place, and (3) standing still. On each type of trial, participants were exposed to 30 s of jittering or smooth radially expanding flow. The display jitter (when present) was generated by incorporating the participant’s own tracked head position (horizontal, vertical, and depth) and orientation (yaw, pitch, and roll) changes directly into the self-motion display (Note: end-to-end system lag measured as ∼60 ms – see Ash et al., 2011 for measurement details). This visually simulated viewpoint jitter was created in real time when participants were actually walking, and was also later played back to them when they viewed these self-motion displays while standing still. During smooth display conditions, these head tracking data were simply ignored and purely radial (i.e., non-jittering), expanding flow displays were presented instead^3^.

During the treadmill walking conditions, the speed of forward self-motion simulated by the radially expanding component of the display matched the speed of the treadmill (Both simulated either 4 or 5 km/h self-motions depending on the trial). By contrast, in the walking in place and the standing still conditions, forward self-motion was only simulated by the display motion (i.e., the treadmill belt was not moving). During the walking conditions (both treadmill walking and walking in place), participants were instructed to try to walk so as to match the speed of their self-motion to that indicated by the radially expanding optic flow display. Two different normal/naturalistic walking speeds were simulated: 4 or 5 km/h. These were deliberately chosen to be close to treadmill belt speeds previously found to enhance optic flow discrimination (Durgin and Gigone, 2007; Durgin, 2009). This rather narrow range of simulated self-motion speeds was also chosen for practical reasons: (1) the acceleration profile of our treadmill was not optimal for slower belt speeds; and (2) our pilot studies indicated that faster belt speeds were too quick for comfortable walking.

Each participant was run through six different blocks of trials. The three different walking types (i.e., treadmill walking, walking in place, and standing still) were examined in separate blocks (with each block being run twice). Four self-motion trials were tested in each block, with the following displays being presented in a fully randomized order: (1) smooth radial flow at 4 km/h, (2) jittering radial flow at 4 km/h, (3) smooth radial flow at 5 km/h, and (4) jittering radial flow at 5 km/h. The order of block presentation was (by necessity) not fully random: One standing still (playback) block was run between two treadmill walking blocks, and the other standing still (playback) block was run between two walking in place blocks^4^.

On all trials, the participant initially held the treadmill’s handrails until he/she was comfortable walking/standing and then released them (only using these again later if needed for support or if he/she became uncomfortable or disoriented). Directly after the 30-s self-motion display, the participant rated the perceived strength of their experience of self-motion for that trial [via a modified version of Stevens’s (1957) method of magnitude estimation]. Each strength rating could range from 0 to 100 (with a rating of “0” indicating no experience of self-motion). These strength ratings were made relative to a standard reference stimulus shown at the beginning of each block of trials. This standard stimulus was a smooth (i.e., non-jittering) pattern of radially expanding optic flow, which simulated forward self-motion at 4 km/h, and was always viewed for 30 s by participants when they were standing still. Participants were told that the strength of the self-motion they experienced during this standard condition corresponded to a rating of “50.” Note that vection (a visually induced illusion of self-motion in a physically stationary observer) could only be potentially induced during standing still conditions (both walking conditions involved whole body motion, even if there was little or no actual displacement relative to the room). However, self-motion was perceived in both walking and stationary conditions – based on multisensory stimulation in the case of the former and visual-only stimulation in the case of the latter.

## RESULTS

### HEAD MOVEMENT ANALYSIS

**FIGURE 2:**
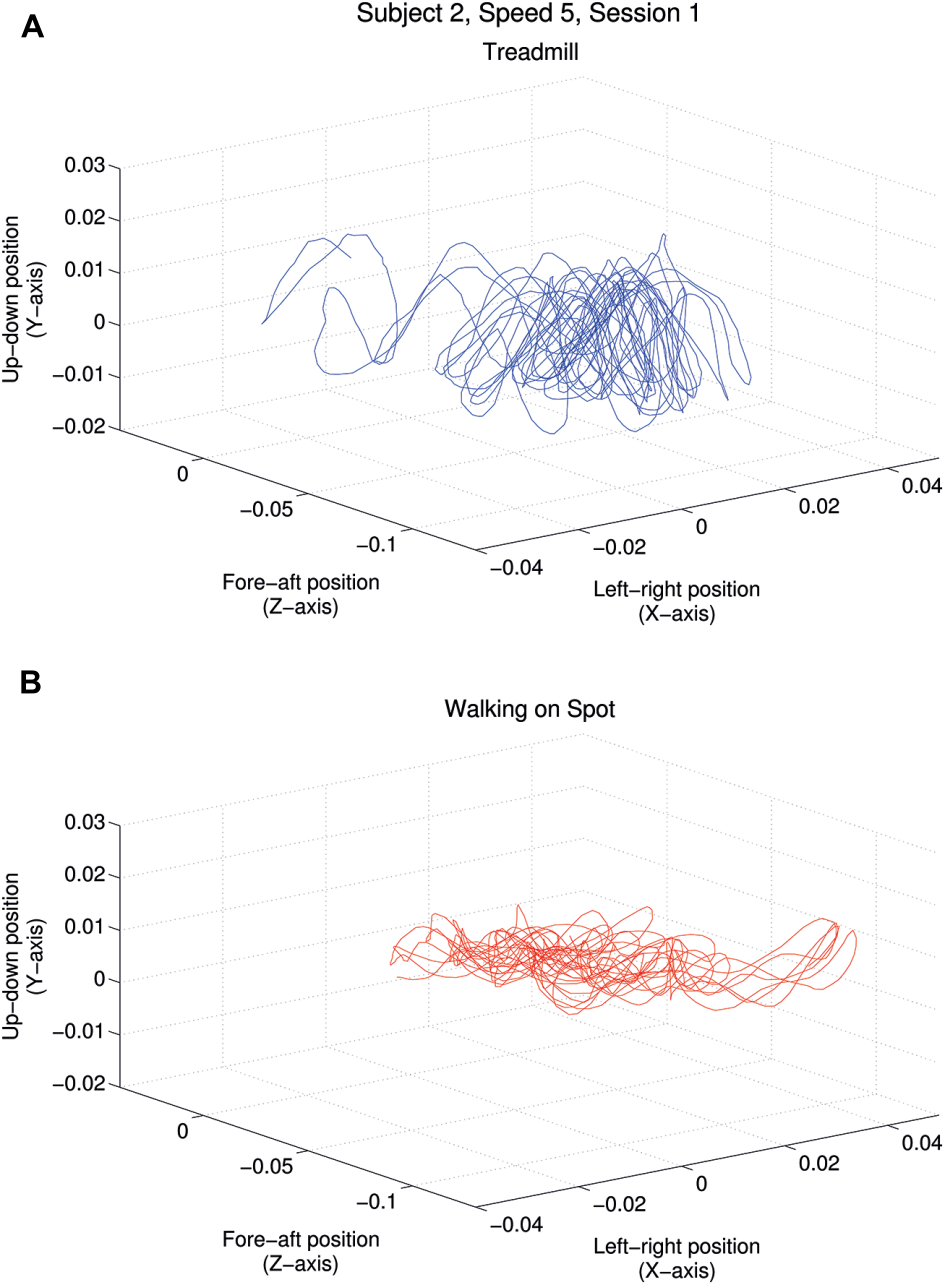
**Horizontal, vertical and depth head position over time for one participant (S2). (A)** Plot shows the head position changes (in meters) of this participant while walking on a treadmill at 4 km/h while viewing a matched speed self-motion display. **(B)** Plot shows head position changes for this participant viewing a similar display (also simulating a 4 km/h forward self-motion) while walking in place.

In the introduction we hypothesized that head jitter amplitudes and frequencies would differ significantly in the “more ecological” treadmill walking and “less ecological” walking in place conditions. Throughout each walking trial, we continuously recorded head position (along the x/horizontal, y/vertical, z/depth axes in m) and head orientation (relative to these same three axes in degrees). In order to test the above hypothesis, we chose to focus only on our participants’ head position changes when walking (because the visual consequences of head position changes could not be nulled by eye movements and were more salient than those resulting from head orientation changes). **Figure 2** provides examples of the raw horizontal, vertical and depth head position changes made during one treadmill walking trial, and one walking in place trial, for one representative participant (S2).

**FIGURE 3:**
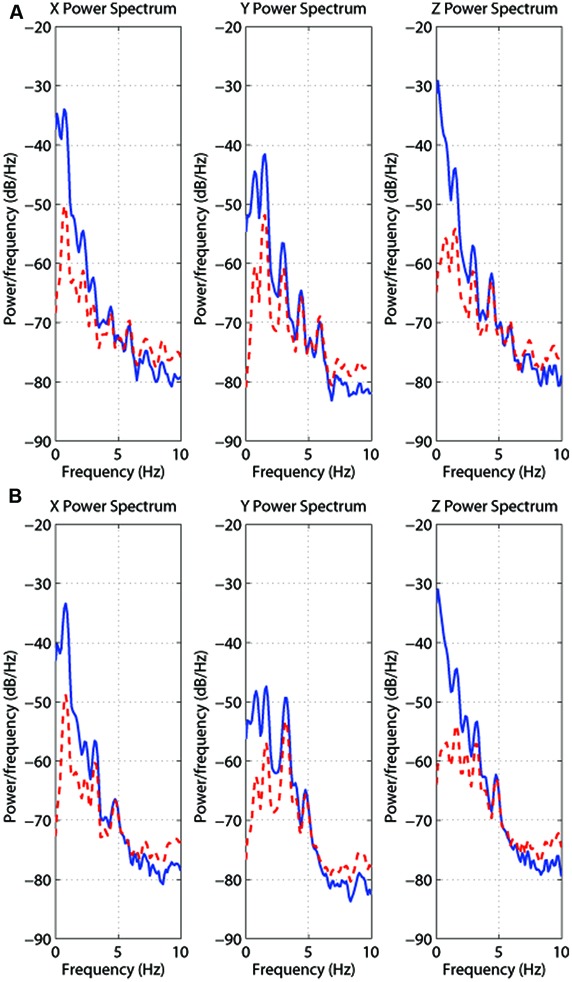
**Power spectrum analyses (blue/unbroken trace = original; red/dashed trace = whitened) of the head position data along each axis for treadmill walking **(A)** and walking in place **(B)**.** These traces are both also for participant S2 here walking at 4 km/h. Note that partial pre-whitening lowers the absolute magnitude of the first peak.

We first performed a power spectrum analysis on this head position data to identify the head movement frequency content during both types of walking condition (i.e., treadmill walking and walking in place). These data were first partially pre-whitened by fitting a first-order autoregressive (AR) model and then filtered with the inverse of this model. This filtering process removed overall broad low-pass characteristics from the data and made the signal whiter, improving the ability to extract the spectral peaks (see Kay, 1988). Then the power spectrum for each trial was estimated using Welch’s (1967) smoothed periodogram and a Hamming window to suppress spectral side lobes. **Figure 3** shows results for one randomly selected participant (S2) during treadmill walking (TOP) and walking in place (BOTTOM). To characterize the spectral composition of the signals, we identified the frequencies and amplitudes of the largest four peaks in the spectrum for each subject and trial type. As the largest peak was typically at least 5 to 10 dB above the next largest peak, we chose to only statistically compare the dominant frequencies and amplitudes of these translational head movements along each axis (these values were likely to be more representative of the subject’s actual cadence).

#### Head movement amplitude

**FIGURE 4:**
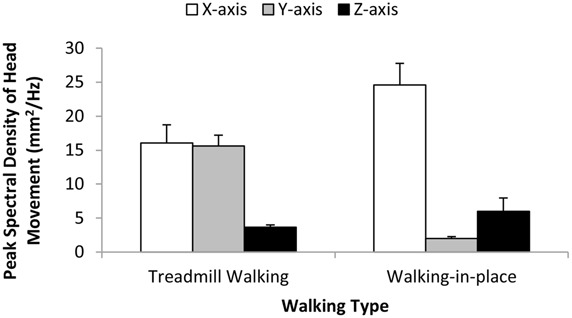
**Effects of *Walking Type* (Treadmill Walking versus Walking in place) on the magnitude of the dominant (pre-whitened) peak head movements along the horizontal (x), vertical (y), and depth (z) axes.** This corresponds to the peak spectral density of the motion signal in each case. Error bars represent SEMs.

***Dominant peak amplitude (individual axes).*** Do the two types of walking produce different dominant peak head movement amplitudes (and if so for which of the three axes)? To answer these questions we performed separate repeated-measures ANOVAs on the magnitude of the dominant peak in the head movement spectra estimated for each axis. The independent variables examined in each of these ANOVAs were walking type (treadmill walking or walking in place) and simulated speed (4 or 5 km/h). We found significant main effects of walking type on dominant x-axis (horizontal) and y-axis (vertical) head amplitudes (*F*_1,18_ = 4.60, *p* < 0.046 and *F*_1,18_ = 98.79, *p* < 0.0001 respectively), but not on dominant z-axis (depth) head amplitudes (*F*_1,18_ = 1.24, *p* = 0.281). Walking in place produced larger x-axis, and smaller y-axis, dominant peak amplitudes than treadmill walking (**Figure 4**). In the case of y-axis dominant peak head movement amplitudes, we also found a significant main effect of simulated speed (*F*_1,18_ = 21.51, *p* < 0.0001) and a significant interaction between simulated speed and walking type (*F*_1,18_ = 18.51, *p* < 0.0001). These findings indicated that y-axis dominant peak head movement amplitudes increased significantly with simulated speed during treadmill walking (but not when walking in place). No other main effects or interactions were found to reach significance.

***Distribution of the spectral content (individual axes).*** The spectral content was distributed quite differently across multiple spectral peaks in the treadmill walking and walking in place conditions. To quantify the spread of power across the peaks, we used the ratio of the magnitude of the second lowest frequency peak to magnitude of the lowest frequency peak. We statistically compared these ratios for head motions along each axis using Bonferroni-corrected contrasts. These ratios were the highest for z-axis head motions, and in fact, were not significantly different for treadmill walking (*M* = 0.43; SD = 0.16) and walking in place (*M* = 0.40; SD = 0.19), *t*_18_ = 0.69, *p*> 0.05. The ratios were, however, significantly lower for y-axis head motions during treadmill walking (*M* = 0.07; SD = 0.05) than during walking in place (*M* = 0.26; SD = 0.18), *t*_18_ = -4.83, *p* < 0.0003. By contrast, the ratios were significantly higher for x-axis head motions during treadmill walking (*M* = 0.17; SD = 0.09) than during walking in place (*M* = 0.06; SD = 0.05), *t*_18_ = 5.16, *p* < 0.0003. These findings suggest that y-axis head motions during treadmill walking (but not during walking in place), and x-axis head motion for walking in place (but not treadmill walking), were each dominated by a single oscillatory motion.

***Head motion energy across axes.*** Head motion energy was observed to be better balanced between horizontal and vertical motion during treadmill walking. Bonferroni-corrected contrasts revealed that: (i) ratios of the dominant x-axis peak motion to the dominant y-axis peak motion were significantly lower for treadmill walking (*M* = 1.1; SD = 1.0) than for walking in place (*M* = 19.3; SD = 20.5), *t*_18_ = -3.97, *p* < 0.002; and (ii) ratios of the dominant y-axis peak motion to the dominant z-axis peak motion were significantly higher for treadmill walking (*M* = 4.9; SD = 3.0) than for walking in place (*M* = 0.73; SD = 0.64), *t*_18_ = 6.26, *p* < 0.0002. Thus, there was a greater bias toward x-axis over y-axis head motion during walking in place and a greater bias toward y-axis over z-axis head motion during treadmill walking.

#### Head movement frequency

**FIGURE 5:**
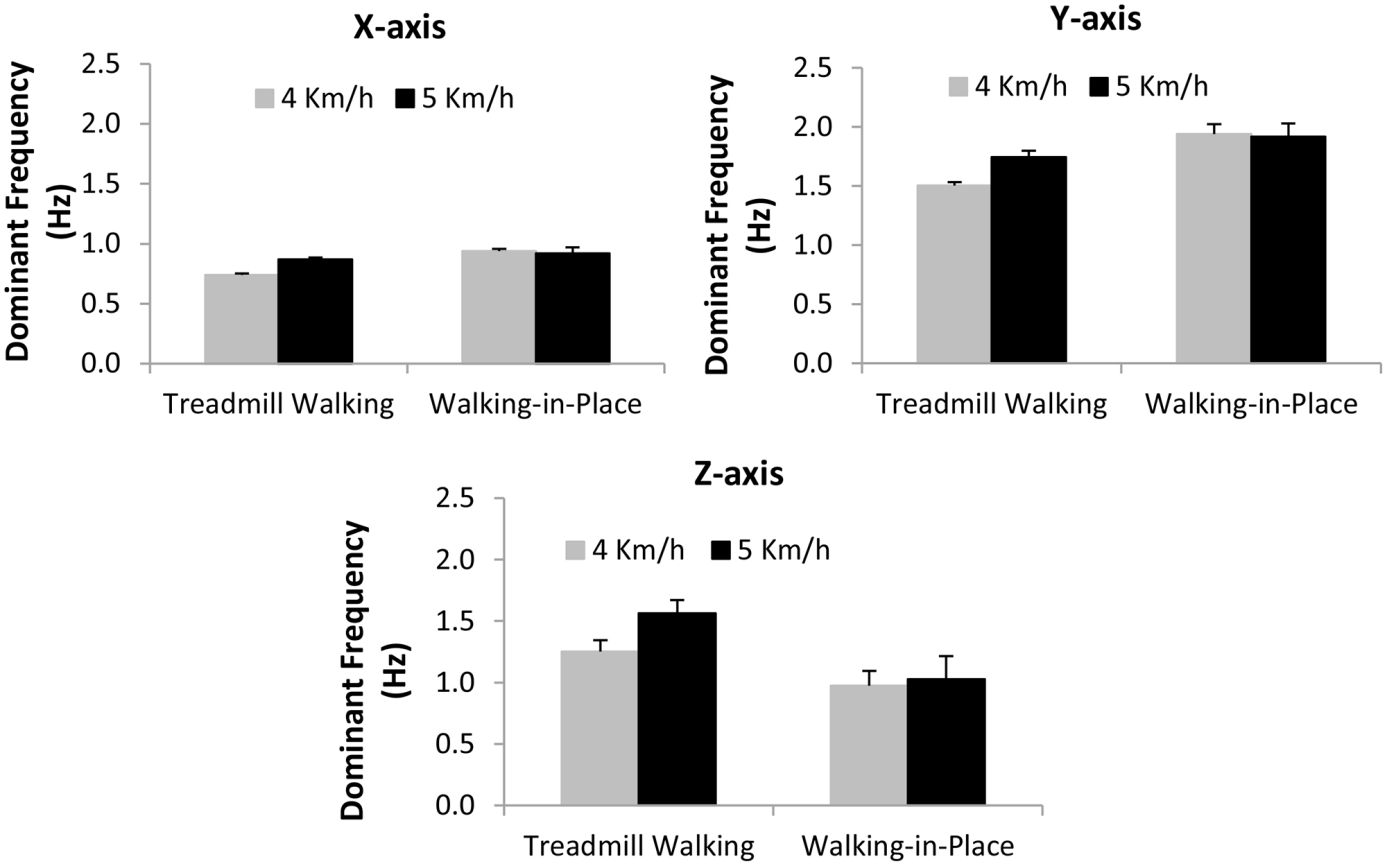
**Effects of *Walking Type* (Treadmill Walking versus Walking in place) and simulated speed (4 versus 5 km/h) on dominant (pre-whitened) peak head movement frequencies (Hz) along the horizontal (x), vertical (y) and depth (z) axes.** Error bars represent SEMs.

We also performed separate repeated-measures ANOVAs on the frequencies of the dominant peaks in the head movement spectra estimated for each of the three axes. The independent variables examined were walking type (treadmill walking or walking in place) and simulated speed (4 or 5 km/h). We found significant main effects of walking type on the frequency of the dominant peak head motion along each of these three axes (x-axis *F*_1,18_ = 31.83, *p* < 0.0001; y-axis *F*_1,18_ = 6.79, *p* < 0.02; z-axis *F*_1,18_ = 8.444, *p* < 0.009). Walking in place generated significantly higher frequency dominant peak x-axis and y-axis head movements, and significantly lower frequency dominant peak z-axis head movements, than treadmill walking (see **Figure 5**). We also found significant interactions between simulated speed and walking type on the dominant peak frequencies of the x-axis (*F*_1,18_ = 77.86, *p* < 0.0001) and y-axis (*F*_1,18_ = 8.91, *p* < 0.008), but not z-axis (*F*_1,18_ = 2.11, *p* < 0.164) head movements (see **Figure 5**). The frequencies of the dominant spectral peaks for both x- and y-axis head motions increased with simulated speed during treadmill walking (by 18 and 16% respectively), whereas they slightly decreased during walking in place (by 2 and 1%). By contrast, the frequencies of the dominant spectral peaks for z-axis head motions increased with simulated speed for both types of walking (by 25% for treadmill walking and 5% for walking in place).

### SELF-MOTION PERCEPTION DATA ANALYSIS

#### Ratings of forward self-motion when standing still (i.e., vection)

**FIGURE 6:**
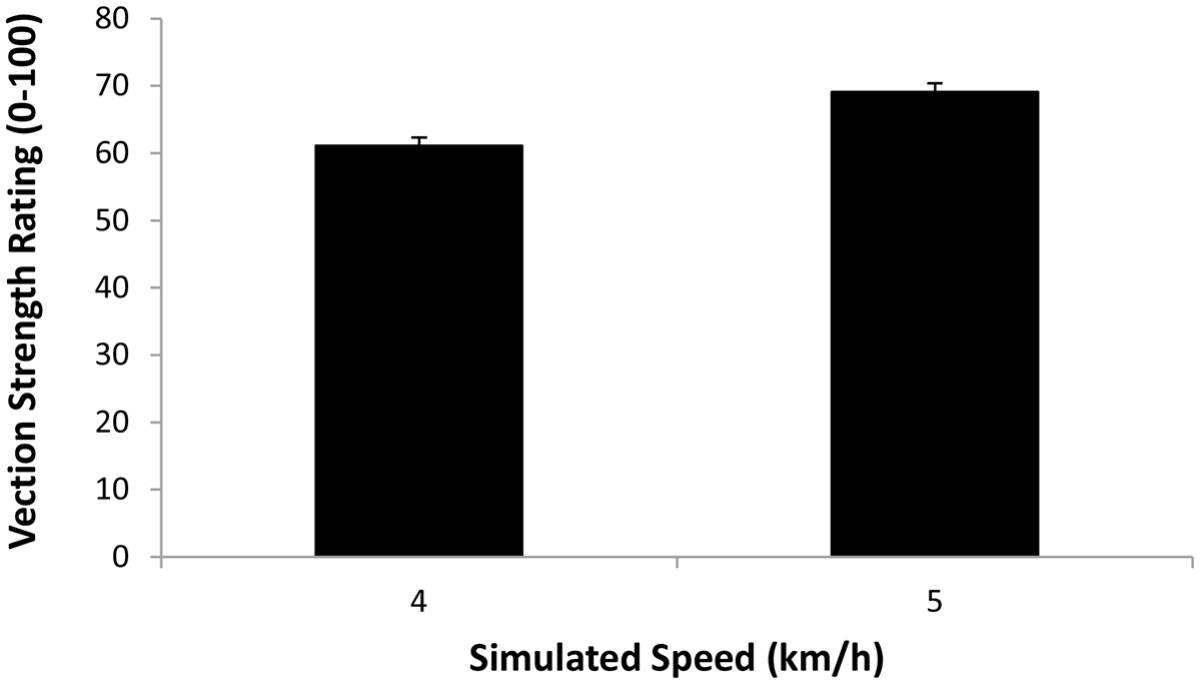
**Effect of *Simulated Speed* (4 or 5 km/h) on the strength of the vection induced when participants were standing still (i.e., *Display Playback*).** Error bars represent SEMs.

**FIGURE 7:**
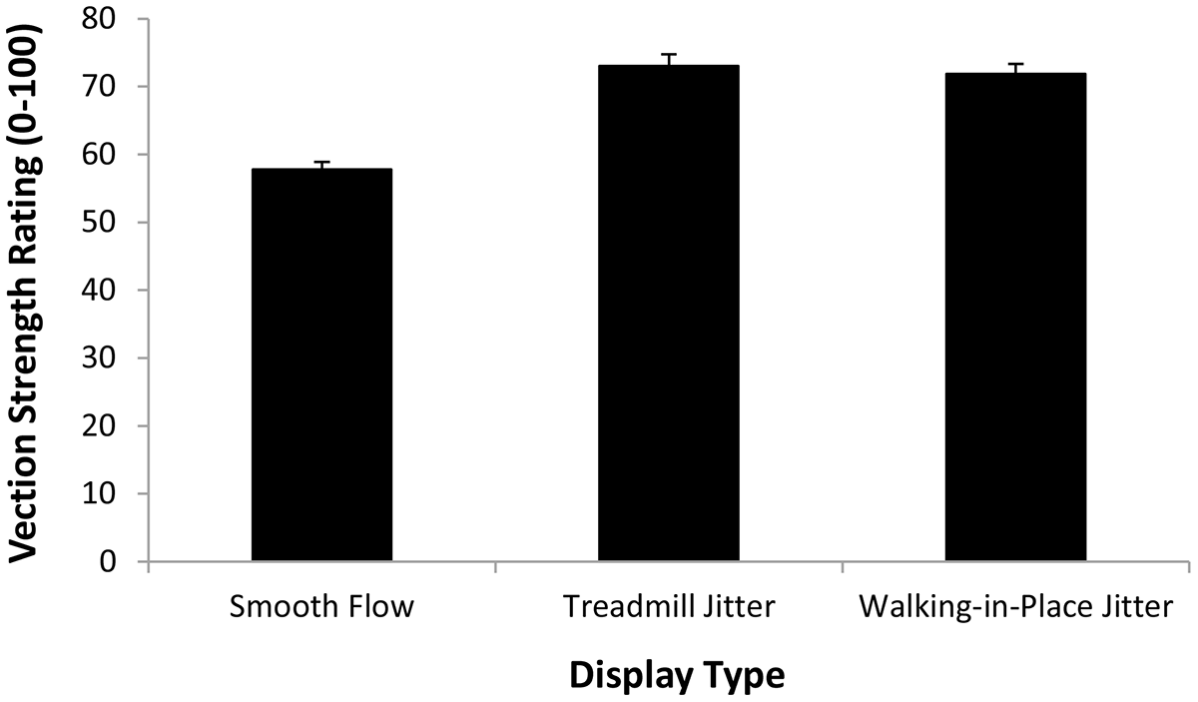
**Effect of *Display Type* (Smooth flow, Treadmill Jitter, and Walking in place Jitter) on the strength of the vection induced when participants were standing still (i.e., *Display Playback*).** Error bars represent SEMs.

The first repeated-measures ANOVA examined the vection strength ratings produced in the display playback conditions (when participants were always standing still). The independent variables examined were display type (smooth, treadmill jitter and walking in place jitter) and simulated speed (4 and 5 km/h). We found a significant main effect of simulated speed (*F*_1,18_ = 97.97, *p* < 0.0001), indicating that displays which simulated 5 km/h self-motions induced stronger vection than those simulating 4 km/h self-motions (see **Figure 6**). We also found a significant main effect of display type (*F*_2,36_ = 29.23, *p* < 0.0001; see **Figure 7**). Bonferroni corrected *post hoc* comparisons showed that: (1) adding either type of jitter (i.e., treadmill walking and walking in place generated jitter) to the radial flow induced significantly stronger vection than the smooth control displays (*p* < 0.05 in each case); and (2) displays with treadmill walking generated jitter did not produce significantly different vection strength ratings to those with walking in place generated jitter (*p* > 0.05). The interaction between display type and simulated speed was also not significant (*F*_2,36_ = 0.877, *p*> 0.05).

#### Ratings of forward self-motion when treadmill walking and walking in place

**FIGURE 8:**
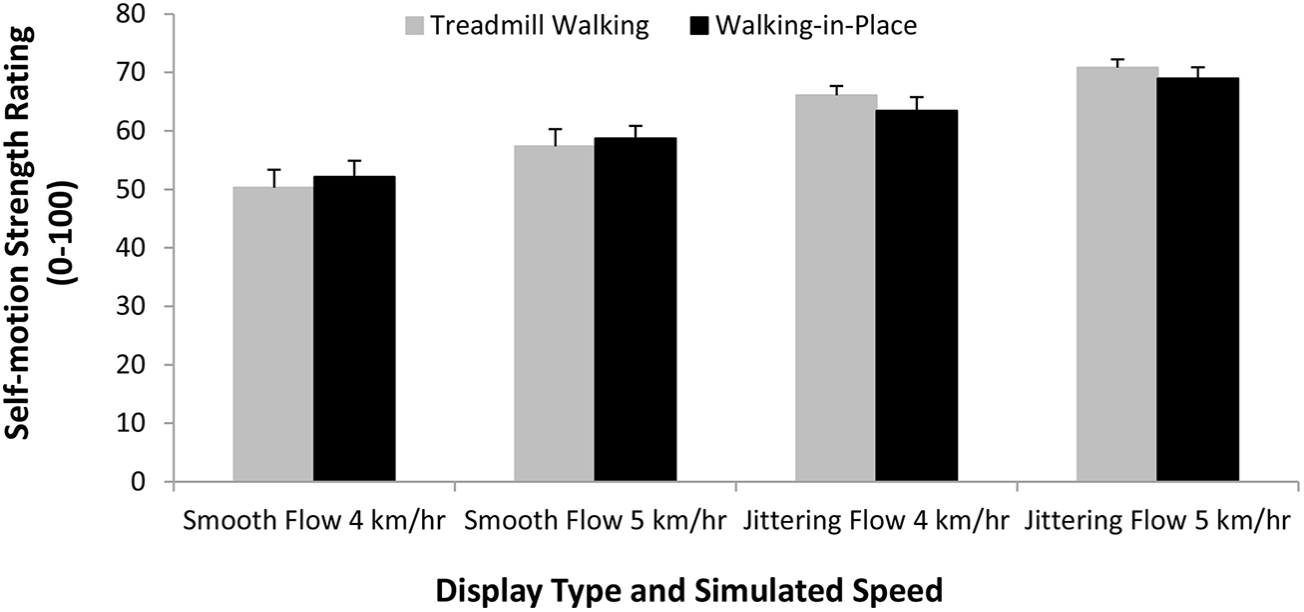
**Effects of *Walking Type* (Treadmill Walking versus Walking in Place), display type (smooth versus jitter) and *Simulated Speed* (4 or 5 km/h) on the strength of the perceived self-motion in depth.** Error bars represent SEMs.

A second repeated-measures ANOVA compared the multisensory perceptions of forward self-motion when participants viewed optic flow displays while treadmill walking and walking in place. The independent variables examined were walking type (treadmill walking and walking in place), display type (smooth and jittering) and simulated speed (4 and 5 km/h). We found no main effect of walking type (*F*_1,18_ = 0.04, *p*> 0.05), which indicated that ratings of the strength of forward self-motion were not significantly different during walking in place and treadmill walking conditions. We also found a significant main effect of simulated speed (*F*_1,18_ = 43.31, *p* < 0.0001), indicating that faster simulated speeds generated stronger ratings of forward self-motion (see **Figure 8**). Importantly we also found a significant main effect of display type (*F*_1,18_ = 25.27, *p* < 0.0001), which indicated that jittering displays produced stronger self-motion ratings than smooth displays (see **Figure 8**). The walking type by display type interaction was not significant (*F*_1,18_ = 1.84, *p*> 0.05), suggesting that treadmill-walking generated jitter and walking in place generated jitter produced very similar advantages/enhancements in terms of the rated experience of self-motion. No other two- or three-way interactions were significant (*p* > 0.05 in all cases).

## DISCUSSION

This study compared the self-motion perceptions induced by treadmill walking and walking in place generated jitter in order to test the ecological account of the jitter advantage for vection. Both types of visual jitter were generated by whole body observer motions, which resulted in complex 6DOF head movements and jittering optic flow. It was predicted that the “more ecological” treadmill walking and “less ecological” walking in place conditions would generate significantly different head (and therefore visual) jitter amplitudes and frequencies. Consistent with this prediction, our head tracking data displayed multiple differences between the two types of walking. Head motion energy was observed to be better balanced between horizontal and vertical motion during treadmill walking. Compared to walking in place, treadmill walking also produced: (1) significantly smaller horizontal, and larger vertical, dominant peak amplitude head jitter; and (2) significantly lower frequency dominant peak horizontal and vertical head jitter, as well as higher frequency dominant peak z-axis head jitter. We also found that the simulated speed manipulation had different effects on the head jitter generated by treadmill walking and walking in place. While increasing the simulated speed of forward self-motion increased the dominant peak amplitude of vertical head jitter as well as the dominant horizontal and vertical head frequency during treadmill walking, it did not alter these during walking in place^5^. It is likely that without the added guidance provided by treadmill belt motion, participants found it difficult to adjust the pace of their walking in place to match the noticeable but rather modest differences in visually simulated speed.

Importantly, the present self-motion strength data provided little support for the ecological account of the jitter advantage for vection. While we found marked differences in the head movement amplitudes and frequencies generated by treadmill walking and walking in place, “more ecological” treadmill walking and “less ecological” walking in place generated jitter produced very similar advantages/enhancements (compared to conditions with smooth, purely radial, optic flow). When participants walked while viewing the computer generated self-motion displays: (1) both types of jitter significantly increased the perceived self-motion in depth induced by the radial component of the flow; and (2) there was no difference between perceptions in the treadmill walking and walking in place conditions. When the jittering self-motion displays recorded during the two types of walking were later played back to stationary observers, the vection advantages produced (again compared to smooth flow control conditions) were virtually identical.

So if visually simulated viewpoint jitter does not improve vection by making the flow appear more ecological, what causes these types of jitter advantages? Currently two explanations have strong empirical support. Adding simulated viewpoint jitter to smooth radial flow might improve vection in depth by: (1) increasing (or perhaps altering^6^) the observer’s overall global retinal motion (Palmisano and Kim, 2009; Palmisano et al., 2012; Nakamura, 2013); and (2) reducing local motion adaptation to the smooth component of the flow (e.g., Seno et al., 2011; Kim and Khuu, 2014). These explanations are not mutually exclusive, and in fact, there appears to be mounting evidence that there may be multiple mechanisms underlying these jitter effects (see Apthorp and Palmisano, 2014). In support of the “increased global motion” explanation, Palmisano et al. (2012) found that the “slalom illusion,” where observers track an oscillating fixation point while viewing smooth radial flow, produced a similar vection advantage to stationary fixation conditions when the display was oscillated. Interestingly, slalom illusion and display oscillation conditions did not produce significantly different motion aftereffects to control conditions with stationary fixation and a smooth non-oscillating display (probably because display durations in all three cases were very short in this study; only 15 s).

In support of the “reduced local motion adaptation” explanation, Kim and Khuu (2014) found reduced motion aftereffects and increased vection in depth for smooth flow when either angular or linear simulated viewpoint oscillation was added. Seno et al. (2011) had earlier found both reduced motion aftereffects and increased vection in depth for linearly jittering and oscillating, compared to smooth, radial flow. Interestingly, they also reported that random jitter, but not oscillation, increased the duration of aftereffects of visual self-motion perception. These vection aftereffects are separate and distinct from general motion aftereffects. Thus, in the Seno et al. (2011) paper, we speculated that these vection aftereffects might reflect the adaptation of a “pure vection mechanism,” over and above lower level motion mechanisms. According to this notion, simulated random viewpoint jitter might have stimulated this pure vection mechanism to a greater extent, while simulated viewpoint oscillation might have tapped into relatively lower-level motion processing.

In the current paper, we utilized complex optic flow patterns containing 6DOF jitter. This walking-generated jitter contained both oscillatory and random components along/about all three axes and thus potentially would have tapped both proposed jitter-enhancement mechanisms (i.e., “increased global motion” and “reduced local motion adaptation” and perhaps others as well).

## CONCLUSION

We have shown that the jitter advantage for visually mediated self-motion perception is very robust. We found a substantial advantage for jittering over smooth self-motion displays, irrespective of whether the participant was walking or standing still when viewing these optic flow displays. Importantly, the advantages found in both scenarios (i.e., when walking or standing still) were remarkably similar for “more ecological” and “less ecological” types of jittering optic flow. Thus, the present data provide little support for the ecological account of these jitter advantages for visually mediated self-motion perception and vection.

## Conflict of Interest Statement

The authors declare that the research was conducted in the absence of any commercial or financial relationships that could be construed as a potential conflict of interest.

## Footnotes

1 One reviewer noted that while walking is arguably the most natural form of self-motion, many passive self-motions (e.g., when in a vehicle) are common in the modern world. These are also accompanied by characteristic patterns of jitter and could thus potentially be deemed ecological as well.

2 While vection is a conscious subjective experience, it is widely regarded to be the result of the very rapid (i.e., preconscious) pickup of optic flow based information (e.g., Warren, 1995; Norman, 2002). Thus, the participant’s ability to consciously discriminate between the different walking conditions was not regarded as a critical test here (since preconsciously detected differences might not be consciously available). It was however essential to show that the physical characteristics of the head movements generated by the more and less ecological walking conditions were in fact significantly different each other.

3 When smooth radial flow displays were presented during walking (as opposed to standing still), they would have visually jittered (due to the relative motion between the walker’s head and the display). While the rotational components of this jitter might have appeared reasonably natural, the linear components contained no motion perspective. Therefore, the visual jitter in this situation would have been incompatible with the 3D environment being simulated by the self-motion display.

4 Prior exposure to optic flow and walking can both alter subsequent vection (e.g., Apthorp and Palmisano, 2014; Seno et al., 2014). Here to control for possible block order effects we averaged the data obtained in the first and last walking blocks together. In order to minimize trial-to-trial carry over effects (i.e., sensory adaptation/readjustment), the minimum inter-trial interval was set at ∼35 s (i.e., longer than the actual duration of the self-motion stimulations).

5 This latter finding is intriguing since research shows that optic flow based speed discriminations are enhanced by treadmill walking at very similar speeds (Durgin and Gigone, 2007; Durgin, 2009). It provides further evidence that walking in place was less ecological than treadmill walking (i.e., the conscious control of walking in place may have bypassed the normal/automatic processing associated with the control of natural walking).

6 Kim and Khuu (2014) have recently suggested that it may not be the overall increase in retinal motion that is important, “but rather, variation in the direction and velocity of visual motion across the retina” (p. 9) which is critical for the jitter advantage for vection.
